# The Influence of Atmospheric Oxygen Content on the Mechanical Properties of Selectively Laser Melted AlSi10Mg TPMS-Based Lattice

**DOI:** 10.3390/ma16010430

**Published:** 2023-01-02

**Authors:** Ahmad Baroutaji, Arun Arjunan, James Beal, John Robinson, Julio Coroado

**Affiliations:** 1 School of Engineering and Applied Science, Aston University, Aston Triangle, Birmingham B4 7ET, UK; 2 Additive Manufacturing of Functional Materials Research Group, Centre for Engineering Innovation and Research, University of Wolverhampton, Telford Innovation Campus, Telford TF2 9NT, UK; 3 School of Engineering, University of Wolverhampton, Telford Innovation Campus, Telford TF2 9NT, UK; 4 Additive Analytics Ltd., Stirchley Road, Telford TF3 1EB, UK; 5 AceOn Battery Solar Technology Ltd., Unit 9B, Stafford Park 12, Telford TF3 3BJ, UK; 6 BOC Ltd., Forge, 43 Church St W, Woking GU21 6HT, UK

**Keywords:** additive manufacturing, selective laser melting, oxygen content, aluminium alloys, AlSi10Mg, TPMS

## Abstract

Selective Laser Melting (SLM) is an emerging Additive Manufacturing (AM) technique for the on-demand fabrication of metal parts. The mechanical properties of Selectively Laser Melted (SLMed) parts are sensitive to oxygen concentration within the SLM build chamber due to the formation of oxides, which may lead to various negative consequences. As such, this work explores the influence of SLM atmospheric Oxygen Content (OC) on the macroscopic mechanical properties of SLMed AlSi10Mg bulk material and Triply Periodic Minimal Surface (TPMS) lattices namely primitive, gyroid, and diamond. Standard quasi-static tensile and crushing tests were conducted to evaluate the bulk properties of AlSi10Mg and the compressive metrics of TPMS-lattices. Two oxygen concentrations of 100 ppm and 1000 were used during the SLM fabrication of the experimental specimens. The tensile test data revealed a small influence of the oxygen content on the bulk properties. The low oxygen concentration improved the elongation while slightly reduced the ultimate tensile strength and yield stress. Similarly, the influence of the oxygen content on the compressive responses of TPMS-lattices was generally limited and primarily depended on their geometrical configuration. This study elucidates the role of SLM atmospheric oxygen content on the macroscopic behaviour of SLMed AlSi10Mg parts.

## 1. Introduction

Additive manufacturing (AM) is commonly referred to as 3D printing; with its unique and significant capabilities, it is debated as a revolutionary step in the paradigm of engineering design and manufacturing. AM enables the production of complex shaped parts from a wide range of materials, which is not achievable via conventional manufacturing methods. AM techniques are increasingly employed to manufacture functional parts in many industrial sectors such as energy [1], automotive [2,3], aerospace [4,5,6], biomedical [7,8,9], and military [10].

Among the various AM techniques, SLM attracted the most attention for the fabrication of metallic parts with relatively small sizes and significant geometrical complexity [11]. SLM is a Powder Bed Fusion (PBF) technology that employs a laser beam to selectively melt the powder particles in the powder bed, enabling the layer-by-layer building of the part. SLM has successfully been used with different metals, such as aluminium, stainless steel, titanium, nickel and tungsten, to produce complex shaped parts for various applications such as biomedical implants, crashworthiness structures, heat exchangers, and so on [12,13,14]. The application of SLM to manufacture aluminium alloys is of particular importance because aluminium is the second most-used metal, after steel, in industry and offers multiple excellent properties such as a low density, high corrosion resistance, high specific strength, and excellent thermal and electric conductivity [15,16].

SLM process involves a series of complex physical and chemical reactions during the melting and solidification of the powder bed, such as powder evaporation, oxidation, laser spatter, etc., which leads to a variety of metallurgical defects such as residual stress, inclusions, porosity, and surface irregularities [12]. Therefore, to achieve a successful and defects-free product using SLM, different material and process parameters, including powder characteristics, laser power, working atmosphere, hatch distance, scan speed, etc., should be controlled and optimised [17,18,19].

As is known, many metals, such as aluminium and titanium alloys, are sensitive to air exposure due to their high affinity to nitrogen and oxygen. Such metals easily react with oxygen and nitrogen, within the air, and form oxides and nitrides, which, in turn, affect their mechanical properties, mainly increasing the strength and reducing the ductility [20]. In the SLM process, high oxygen content in the processing environment or powder may yield various metallurgical defects, such as inclusions, porosity, and micro-cracks, which can negatively impact the density and mechanical properties of the part [21,22]. Therefore, a controlled inert gas atmosphere is used during the SLM process to limit the oxygen and nitrogen pick-ups by the built material, minimize the formation of oxides and nitrides phases, improve the formability, and enhance the mechanical properties of the final product. The inert gas not only affects the final product but also has an impact on the stability and build rate of the SLM process itself [20].

The influence of atmospheric oxygen content on the mechanical properties and defects of SLMed metals has been explored in some research studies. Rombouts et al. [23] found that the high content of atmospheric oxygen in the build chamber during SLM of alloyed steel increases the melt pool volume during SLM, which consequently leads to the formation of large balling droplets in the final product, reducing its surface quality. Li et al. [24] controlled the level of atmospheric oxygen content during the SLM of stainless steel and stated that atmospheric oxygen plays a key role in the initiation of the balling phenomenon, which can be reduced by maintaining low oxygen content in the atmosphere. Dietrich et al. [25] studied the influence of oxygen content on the chemical composition and mechanical properties of SLMed Ti-6Al-4V. It was found that increasing the atmospheric oxygen content leads to an increase in the ultimate tensile strength and decrease in the fatigue resistance. Pauzon et al. [26] investigated the effect of oxygen concentration in the SLM build space on Ti-6Al-4V. It was found that increasing the levels of oxygen and nitrogen in the process atmosphere leads to an increase in the oxygen and nitrogen content in the built material, which, in turn, causes significant embrittlement in the built materials, increasing its strength and decreasing its elongation. Hu et al. [27] examined the influence of atmospheric oxygen content on the fracture behaviour and mechanical properties of SLM-fabricated AlCu5MnCdVA material. It was found that the samples built with a low oxygen content of 20 ppm in the build chamber have a greater tensile strength and higher elongation than their counterparts prepared with a higher oxygen content of 200 ppm. Iveković et al. [28] changed the oxygen level in the build chamber during the SLM process of pure tungsten and tungsten alloys and found that the low oxygen content results in parts with a higher density. From the literature that was surveyed earlier, the influence of oxygen content on the SLM process of aluminium alloy, particularly AlSi10Mg, has received limited attention.

Lattice structures, or metamaterial, are artificial cellular materials that offer adjustable and tuneable performance based on the topology of a sub-structure called a unit cell [13,29,30]. They enable unprecedented characteristics, such as lightweight, negative Poisson’s ratio, high strength-to-weight, etc., which are hard to realize in conventional natural materials. Due to their potential, novel mechanical properties, and advanced functionalities, developing metamaterials is a hot research topic, which attracted a good deal of effort for a wide range of engineering applications. The development of innovative metamaterials is closely linked with the rapid advances in the AM methods that enabled the fabrication of lattices with extremely complex unit cells, such as TPMS surfaces. The TPMS-lattices provide a superior mechanical performance compared to their strut-based counterparts. Additionally, TPMS-lattices, with their advanced surface features, such as uniform curvature radius and smooth surface, exhibit better AM manufacturability than strut-based lattices due to their self-supporting characteristics [31]. Therefore, such lattices were the subject of many studies in the literature. Al-Ketan et al. [32,33,34] comprehensively investigated the mechanical properties of different TPMS-lattices with different unit cell architectures, such as gyroid, diamond, primitive, etc., under quasi-static compressive loading. Novak et al. [35] investigated the quasi-static and dynamic responses of TPMS-lattices made of 316 L stainless steel via SLM. Novak et al. [36] investigated the blast and impact resistance of sandwich panels containing uniform and graded TPMS-based cores. TPMS-lattices manufactured from lightweight metal alloys, such as aluminium alloy, are excellent candidates for lightweight energy-absorbing applications in aerospace and automotive industries as well as personal protective equipment; thus, they have attracted significant interest from the research community. Hou et al. [37] studied the damage failure of AlSi10Mg TPMS porous structures under quasi-static and dynamic loading cases. Wu et al. [38] developed a finite element (FE) model to analyse the mechanical and energy absorption responses of the AlSi10Mg gyroid TPMS lattice. Yang et al. [31] used the FE method to predict the mechanical properties and failure mechanisms of the TPMS gyroid lattice under compression loading. Ejeh et al. [39] explored the flexural properties of a laser-melted graded TPMS latticed beam made of AlSi10Mg.

The research investigations on the SLMed TPMS-lattices have mainly focused on the design and mechanical behaviour, while the effects of SLM process parameters on the responses of such structures have not been explored to date. Thus, the main aim of the current paper is to investigate the influence of atmospheric oxygen concentration on the bulk mechanical properties of AlSi10Mg as well as the quasi-static mechanical performance of SLMed AlSi10Mg TPMS-lattices. Three different geometries of TPMS-lattices including primitive, gyroid, and diamond were analysed. All the samples were fabricated using a selective laser melting system with controlled oxygen content in the build chamber. Tensile and compression tests were conducted to extract the mechanical properties of the studied configuration and to analyse the effect of oxygen concentration.

## 2. Methodology

### 2.1. TPMS-Lattice Design

Triply Periodic Minimal Surface (TPMS) has a zero-mean curvature at any of its points and it is periodic in the three independent directions *x, y,* and *z* [33,40]. The geometrical shape of such surfaces is a continuous and non-intersecting surface dividing the space into two sub-domains. Such surfaces can be expressed mathematically using an implicit method, known as level-set approximation, that contains a combination of trigonometric functions. The general form of the level-set equation is shown in Equation (1)

(1) $$\emptyset_{TPMS}\left(x,y,z\right)=c$$

where c is the level-set constant. By adjusting the value of *c*, one can control the surface area of the TPMS as well as the volume of the two subdomains bounded by the TPMS [36]. The two sub-domains have equal volumes when c=0, while they will have different volumes when c≠0. The TPMS-lattice can be obtained by offsetting the TPMS in two directions and making the volume enclosed by the resulting surfaces solid. The level-set equation for TPMS-lattice is given in Equation (2)

(2) $$-c\leq \emptyset_{TPMS}\left(x,y,z\right)\leq c$$

In the current work, three different TPMS surfaces, including Primitive (TPMS-P), Gyroid (TPMS-G), and Diamond (TPMS-D), were considered. The mathematical expressions of TPMS-P, TPMS-G, and TPMS-D are shown in Equations (2)–(5)

(3) $$\emptyset_{TPMS-P}\left(x,y,z\right)=\cos\left(\omega x\right)+\cos\left(\omega y\right)+\cos\left(\omega z\right)=\pm t$$

(4) $$\emptyset_{TPMS-G}\left(x,y,z\right)=\sin\left(\omega x\right)\cos\left(\omega y\right)+\sin\left(\omega y\right)\cos\left(\omega z\right)+\sin\left(\omega z\right)\cos\left(\omega x\right)=\pm t$$

(5) $$\emptyset_{TPMS-D}\left(x,y,z\right)=\cos\left(\omega x\right)\cos\left(\omega y\right)\cos\left(\omega z\right)+\sin\left(\omega x\right)\sin\left(\omega y\right)\sin\left(\omega z\right)=\pm t$$

where *x, y, z* are spatial coordinates, ω=2π/l in which *l* represents the length of a unit cell, *t* is the level-set constant of the TPMS surface such that changing the value of *t* will change the thickness of the TPMS-lattice and, hence, its relative density [39].

**Figure 1:**
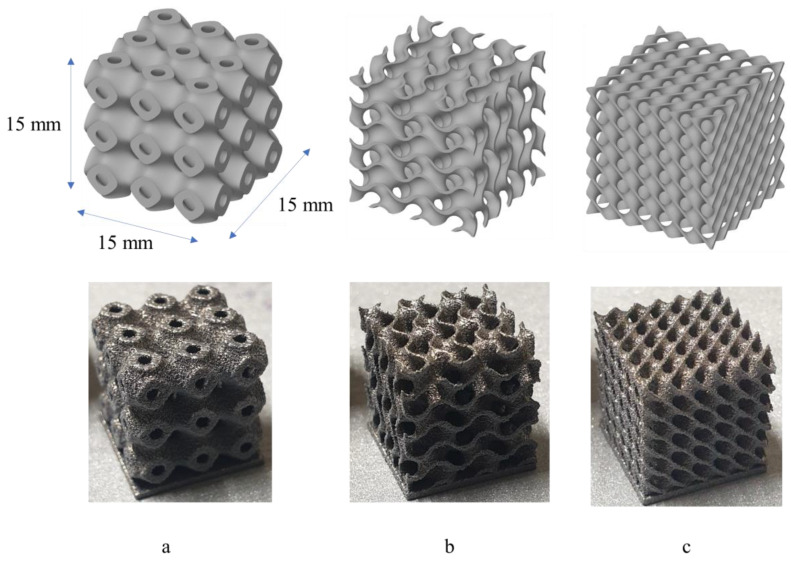
TPMS-lattices: (**a**) TPMS-P (**b**) TPMS-G (**c**) TPMS-D.

The TPMS-lattices were produced with three-unit cells along x, y, and z directions. The unit cell size is 5 mm, resulting in a global size of 15 mm × 15 mm × 15 mm, as shown in Figure 1. The CAD models of the TPMS-lattices were generated using nTopology and then exported as Stl. to be processed by the additive manufacturing system. The TPMS samples were fabricated with different thicknesses to evaluate the influence of OC on the samples when they have different geometrical dimensions. The wall thickness of TPMS-G and TPMS-D is 0.3 mm, while the thickness of TPMS-P is 0.1 mm.

### 2.2. Fabrication

**Figure 2:**
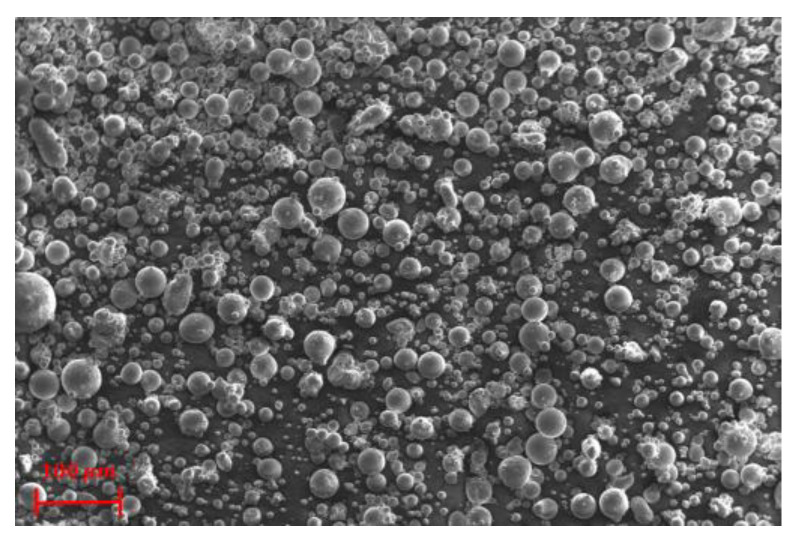
SEM micrograph of AlSi10Mg powder feedstock used for SLM.

Gas-atomized AlSi10Mg aluminium alloy powder was used to fabricate the different specimens, i.e., TPMS-lattices, and the tensile samples. The AlSi10Mg powder particles have a nearly spherical morphology, smooth surface, and smaller particle size than 90 μm, as can be seen in Figure 2. The material composition of AlSi10Mg is given in Table 1.

A commercial EOS M 290 machine (EOS GmbH) was used as the SLM printer. ADDvance^®^O₂ precision system (Linde GmbH) was connected to the EOS M 290 and used to precisely control and monitor the oxygen content in the process atmosphere by regulating the inert gas environment within the build chamber. An inert argon environment was used in the building chamber. Two levels of atmospheric oxygen concentrations, including 100 ppm and 1000 ppm, were used. Prior to SLM, the build chamber was purged with argon until the desired oxygen content was reached, and then the gas exchange valve in the chamber was switched off to maintain a constant concentration of oxygen. The atmospheric oxygen content within the SLM build chamber was continuously monitored by oxygen sensors and ensured to be at the set value.

The parts were built layer-by-layer, where the powder feeder spread the powder over the build platform, and then the laser beam melted the powder in specific areas of the powder bed according to the CAD model of the part. The building platform was lowered after completing each layer, where the process of spreading and melting the powder was repeated until the part was finished. The SLM system was set with 370 W, 1300 mm/s, 0.19 mm, and 30 μm as laser power, scan speed, hatch distance, and layer thickness, respectively. These parameters were recommended by the manufacturer to achieve highly dense prints (approx. 99.8%) from AlSi10Mg powder [18,41]. The SLM-built parts were heat-treated at 300 °C for 2 h in an air atmosphere, and then they were removed from the building platform using the Wire Electric Discharge Machining (WEDM) system.

### 2.3. Tensile and Compression Tests

The bulk mechanical properties of the AlSi10Mg were extracted using standard uniaxial tensile tests on flat dog-bone specimens according to BSEN ISO 6892-1 standards [42]. The specimens have a gauge length of 35 mm and a thickness of 5.4 mm. The tensile tests were conducted at room temperature with a loading rate of 4 mm/min.

The mechanical properties of the TPMS-lattices were extracted by carrying out uniaxial quasi-static compression tests following BSEN ISO 13314 standards [43]. Each sample was crushed between a fixed upper base and a lower moving base. The samples were placed in the centre of the loading base to ensure uniform axial loading. The tests were displacement-controlled with a loading rate of 4 mm/min. Each TPMS sample was compressed by at least 12 mm, approximately 80% of its length, and the tests were stopped when either the force threshold or sample densification was reached to avoid any possible damage to the testing kit.

Both tensile and compression tests were conducted using the Zwick-1474 system. This system uses a load cell with a maximum capacity of 100 kN to measure the forces acting on the sample during the testing. Before conducting the experimental tests, the rig was calibrated according to BSEN ISO 7500-1 standard [44]. A high-speed Cannon DSLR camera was used to monitor the deformation behaviour of the TPMS samples whilst the load and displacement data were recorded by the Zwick-1474 system and then exported to excel files for further analysis. It is worth noting that two specimens of each TPMS-lattice and tensile part under each oxygen content were fabricated and tested to ensure the reproducibility and reliability of the experimental results.

### 2.4. Mechanical Properties of TPMS-Lattice

**Figure 3:**
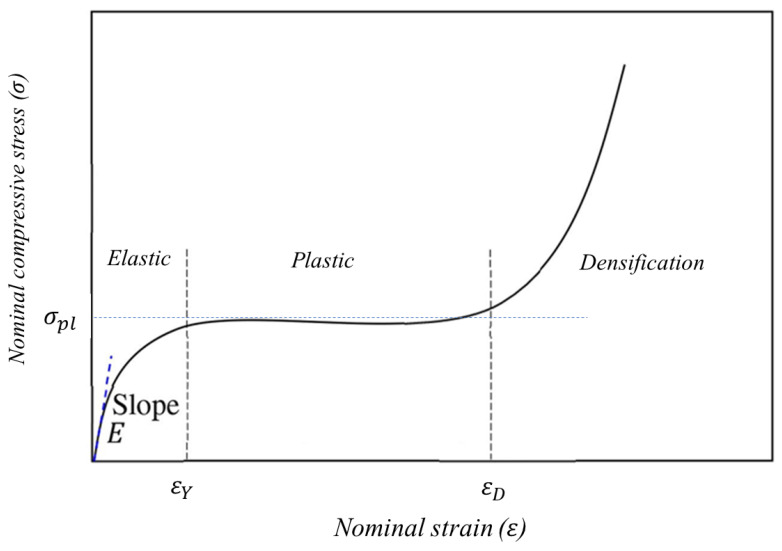
Typical stress–strain response of a lattice structure subjected to quasi-static compressive loading [45].

In this work, the main mechanical properties, including Young’s modulus (E), yield stress $\left(\sigma_{Y}\right)$, peak stress $\left(\sigma_{Peak}\right)$, plateau stress $\left(\sigma_{Pl}\right)$, toughness (U), and specific energy absorption (SEA) of the TPMS-lattices were extracted from the stress–strain responses. The typical stress–strain characteristic of a lattice structure under compressive loading consists of three distinct stages including elastic, plateau, and densification, as shown in Figure 3.

E can be determined as the slope of the linear part of the stress–strain curve. $\sigma_{Peak}$ is the maximum stress observed in the range up to the densification strain ($\varepsilon_{D}$). The plateau stress is the average stress between the yielding and densification strains, and can be calculated using Equation (6)

(6) $$\sigma_{Pl}=\frac{\int_{\varepsilon_{Y}}^{\varepsilon_{D}}\sigma \left(\varepsilon \right)d\varepsilon }{\varepsilon_{D}-\varepsilon_{Y}}$$

The 0.2% offset method was used to determine the yielding point properties, i.e., $\sigma_{Y}\;and\;\varepsilon_{Y}$, while the Gibson–Ashby densification strain relation is employed to estimate the densification strain $\varepsilon_{D}$. The Gibson–Ashby relation uses a linear function between the densification strain and the relative density ($\rho_{r}$) of the lattice [38], as shown in Equation (7)

(7) $$\varepsilon_{D}=1-\alpha \left(\rho_{r}\right)$$

where α varies between 1.4 and 2. α=1.4 was used in this paper. $\rho_{r}$ is the ratio between the density of the lattice ($\rho_{L}$) and the density of the base material ($\rho_{m}$) as given in Equation (8)

(8) $$\rho_{r}=\frac{\rho_{L}}{\rho_{m}}$$

The toughness (U) is another important mechanical property referring to the amount of energy absorbed by the lattice per its unit volume. U can be obtained as the area under the stress–strain curve up to the densification strain [36]; and it can be computed according to Equation (9)

(9) $$U=\int_{0}^{\varepsilon_{D}}\sigma \left(\varepsilon \right)d\varepsilon$$

Finally, Specific Energy Absorption (SEA) was also calculated as another indicator of TPMS-lattice’s ability to absorb energy. SEA is the energy absorbed per unit mass of lattice and can be obtained by dividing the toughness by the density of the lattice as shown in Equation (10)

(10) $$\mathrm{SEA}=\frac{U}{\rho_{L}}$$

## 3. Results and Discussion

### 3.1. Influence of OC on Bulk Properties

**Figure 4:**
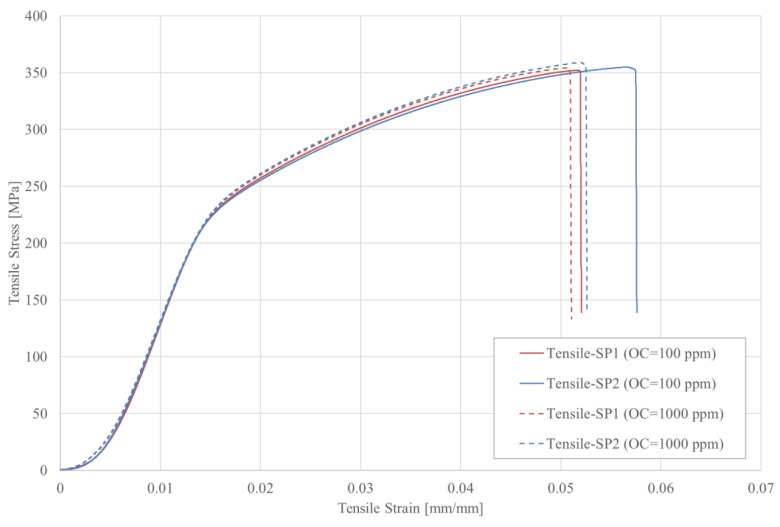
Tensile stress–strain curves of SLMed AlSi10Mg fabricated with different SLM atmospheric oxygen content.

Figure 4 presents the tensile stress–strain curves while Table 2 summarizes the bulk mechanical properties of SLMed AlSi10Mg. The data presented in Table 2 are the mean of the values obtained at each oxygen content. The stress–strain curves almost overlap in the elastic region indicating that the atmospheric oxygen content has a small effect on the elastic properties of the samples. This is also confirmed by the calculated Young’s modulus (Table 2), which is almost constant for both sets of oxygen contents. With decreasing the OC from 1000 ppm to 100 ppm, the strain to failure ($\varepsilon_{fail}$) increased from 0.052 to 0.055. The overall increase in $\varepsilon_{fail}$ is ~5.7%. Both yield stress ($\sigma_{Y}$) and ultimate tensile strength ($\sigma_{UTS}$) decreased with decreasing oxygen concentration in the build chamber. However, the decrease in the values of these properties is very small and does not exceed 3% and 1% for $\sigma_{Y}$ and $\sigma_{UTS}$, respectively. Overall, these results indicate that the impact of the oxygen content on SLMed AlSi10Mg is insignificant. These findings agree well with previous literature studies. Fiedler et al. [46] found that SLM atmospheric oxygen content in the range up to 800 ppm has no influence on the hardness and microstructure of SLMed AlSi10Mg.

### 3.2. Influence of OC on Mechanical Properties of TPMS-Lattice

This section discusses the stress–strain responses, deformation modes, and mechanical properties for all TPMS-lattices investigated in this work. Two specimens of each TPMS-lattice were crushed until the densification stage was reached and a sharp increase was observedin the stress response. The average mechanical properties were extracted from the two stress–strain responses for each lattice.

**Figure 5:**
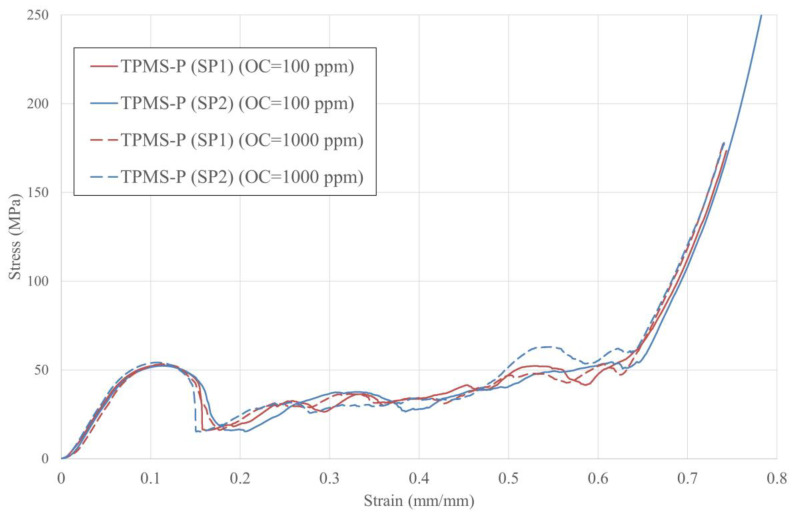
Stress–strain response of TPMS-P.

**Figure 6:**
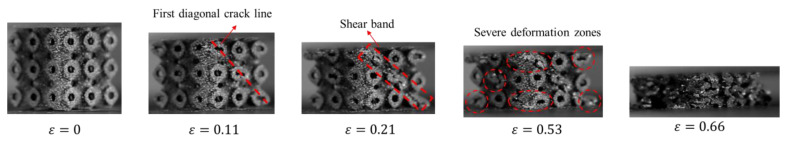
Deformation mode of TPMS-P.

The stress–strain relations and deformation behaviour of TPMS-P lattices are presented in Figure 5. As can be seen, the stresses increase rapidly in the elastic range and then drop sharply when the samples start to yield triggering the start of the plateau regime. This significant decrease in the stress value accounts for ~70% degradation in the mechanical strength of the TPMS-P sample. The softening behaviour observed at this mark, i.e., after the first peak, is due to the formation of an abrupt, diagonal, corner-to-corner crack line, i.e., shear band, where the two neighbouring halves of the sample split and slip with a low-strength ability, as seen in Figure 6. The formation of a shear band during the collapse of primitive TPMS lattice has also been observed in previous investigations [32,47]. In the plateau stage, the stress gradually increases with small fluctuations until the densification point is reached, after which the stresses sharply increase again. The deformation pattern in this stage is a combination of sliding along the shear plane, i.e., diagonal line, and localised crushing within its vicinity. With the continuation of the compression process, the severe deformation spread through the lattice to other zones (see Figure 6). The densification stage starts at the ~0.6 strain, where all the cells collapse completely, their walls start to contact each other, and the lattice starts to behave like a fully dense solid material.

It is clear from Figure 5 that the stress–strain curves of all TPMS-P samples are similar, indicating a small impact of the SLM oxygen content on the mechanical responses of these samples. To quantify the influence of OC, the mechanical properties of TPMS-P samples were calculated, averaged, and presented in Table 3 as functions of the SLM atmospheric oxygen content. Table 3 clearly shows that the mechanical properties of TPMS-P at the two levels of oxygen are almost the same. $\sigma_{Peak}$ and $\sigma_{Y}$ of the TPMS-P samples fabricated with OC of 100 ppm is 8% less than those samples printed with OC of 1000 ppm. The other mechanical properties exhibit negligible changes with the level of oxygen concentration.

**Figure 7:**
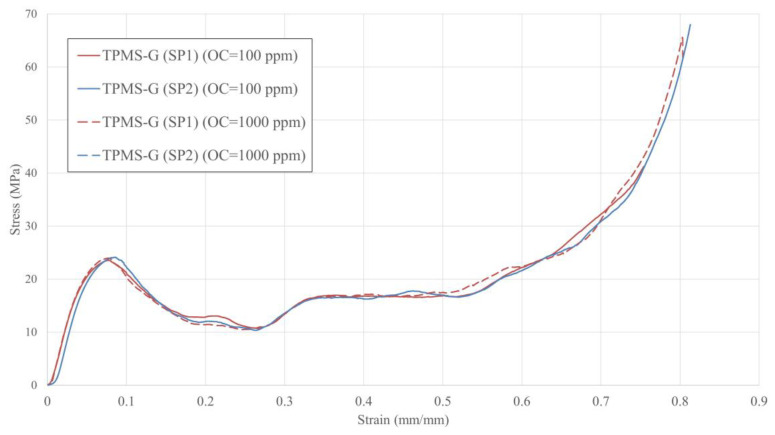
Stress–strain response of TPMS-G.

**Figure 8:**
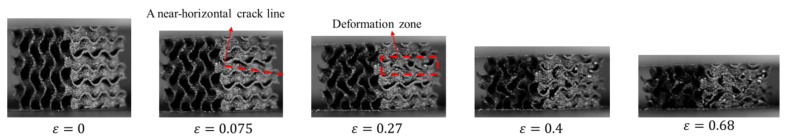
Deformation mode of TPMS-G.

Figure 7 and Figure 8, and Table 4 detail the stress–strain responses, deformation patterns, and mechanical properties of TMPS-G lattices. These samples display the first peak stress of ~24 MPa at ~0.075 strain, followed by a gradual strain-softening behaviour between 0.075 and 0.27 strains. Upon reaching the first stress peak, the collapse initiates in the layers close to the middle region of the lattice, where a near-horizontal crack line is generated and the severe deformation is localised in the cells located around this line (Figure 8). With increasing the compression distance, the other layers of the lattice gradually start to collapse. The plateau region shows almost a steady, i.e., flat, stress–strain response without any stress fluctuations but with slight strain-hardening behaviour. This behaviour is due to the absence of an abrupt shear band, leading to the uniform and simultaneous deformation of its layers. Similar observations have also been reported in other studies [40,48]. Zhang et al. [48] noticed that the layers of a uniform gyroid lattice simultaneously deform during the compression process. The plateau stage continues until a strain value of 0.68, where local densification starts to take place, accompanied by a significant increase in the slope of the stress response.

Similar to TPMS-P, the stress–strain responses of TPMS-G, show almost small to zero change with the level of OC. According to Table 4, the TPMS-G samples fabricated with OC of 100 ppm show average yield stress of 19.15 Mpa, which is 23% higher than their counterparts prepared with OC of 1000 ppm. The OC has also influenced Young’s modulus of the TPMS-G samples, where E decreased by almost 6% by decreasing OC from 1000 ppm to 100 ppm. The other mechanical properties show very small variations with changes in the OC and remain almost constant.

**Figure 9:**
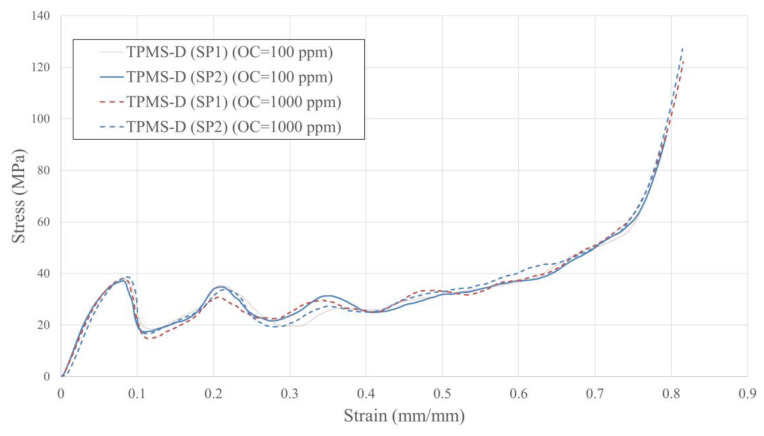
Stress–strain response of TPMS-D.

**Figure 10:**
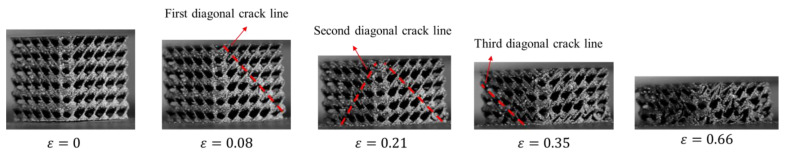
Deformation mode of TPMS-D.

The experimental stress–strain characteristics and the deformation history of TPMS-D specimens are shown in Figure 9 and Figure 10, respectively. Firstly, the stresses linearly increase in the elastic region to ~30 MPa and then continue to increase nonlinearly to a peak value of ~37 MPa. Right after the peak stress is reached, a diagonal crack line is formed (Figure 10), where the lattice collapses by splitting and sliding along this crack line, leading to a steep decrease in the stress values, as can be observed at ~0.8 strains. This steep decrease in the stress value marks the start of the plateau stage. The stress in the plateau region exhibits multiple peaks and gradually increases, with the strain showing an overall strain-hardening behaviour. The multiple peaks observed in this design are due to the formation of additional shear bands on the faces adjacent to the face where the initial crack is formed, as can be seen in Figure 10. The plateau stage continues until a strain value of ~0.55; after which, the stresses sharply increase, indicating the start of the densification stage.

The overall stress–strain responses of the TPMS-D lattices are very comparable, revealing a minimum influence of the oxygen content on the compressive behaviour of the TPMS-D samples. The mechanical properties of TPMS-D structures deduced from the stress–strain responses are tabulated in Table 5. A very slight increase in the material toughness, plateau stress, and specific energy absorption is noted for the samples fabricated with a low OC. However, this increase is less than 2% and, therefore, it can be concluded that the mechanical properties of TPMS-D structures remain unchanged when changing the OC.

## 4. Conclusions

This study investigated the role of SLM atmospheric oxygen concentration on the mechanical performance of laser-melted AlSi10Mg bulk material and TPMS-lattices. The experimental samples were fabricated at two atmospheric oxygen contents of 100 ppm and 1000 ppm and were characterised by their tensile and compressive behaviour. The main findings of the current research can be summarised as follows:

- The SLM atmospheric oxygen content has a limited effect on the bulk properties of AlSi10Mg. Lowering the oxygen content from 1000 ppm to 100 ppm only yielded an increase of ~5.7% in the material ductility, and a decrease of 3% and 1% in its ultimate tensile strength and yield stress, respectively.
- Similar to the bulk properties, the influence of the atmospheric oxygen concentration on the mechanical responses of the TPMS-lattices was found to be low but dependent on the geometrical shape of the lattice.
- For the diamond TPMS-lattices, the low oxygen content offered an increase of <2% in their material toughness, plateau stress, and specific energy absorption.
- For the primitive TPMS-lattices, the strength and yield stress decreased by ~8% when the oxygen content was decreased to 100 ppm.
- For the TPMS with gyroid configuration, the yield stress increased while Young’s modulus decreased when decreasing the oxygen content.

The current work is a preliminary investigation into the influence of the oxygen content, in the range from 100 ppm to 1000 ppm, on the macroscopic performance of SLMed AlSi10Mg parts. To reveal the full impact of the SLM atmospheric oxygen content, further research works are required, where a wider range of the oxygen content (such as 20 ppm to 2000 ppm) can be considered, along with an evaluation of the influence of oxygen content on the microstructure and surface morphology of the manufactured parts.

## Figures and Tables

**Table 1:** Chemical composition of AlSi10Mg (wt. %).

AlSi10Mg										
Al	Si	Fe	Cu	Mn	Mg	Ni	Zn	Pb	Sn	Ti
Bal.	9–11	0.055	0.05	0.45	0.2–0.45	0.05	0.1	0.05	0.05	0.15

**Table 2:** Bulk properties of SLMed AlSi10Mg.

OC (ppm)	E (GPa)	$\sigma_{Y}$ (MPa)	$\sigma_{UTS}$ (MPa)	$\varepsilon_{fail}$ (mm/mm)
100	22.75	238	353.37	0.055
1000	22.016	244	356.46	0.052

**Table 3:** Mechanical properties of TPMS-P.

OC (ppm)	$\rho_{r}$	$\sigma_{Peak}$ (MPa)	$\sigma_{Y}$ (MPa)	$\sigma_{Pl}\;$ (MPa)	E (MPa)	U (kJ/m^3^)	SEA (kJ/kg)
100	0.3307	50.37	38.95	35.96	807.88	18,206	20.62
1000	0.3307	55.12	42.35	36.22	807.91	18,319.50	20.75

**Table 4:** Mechanical properties of TPMS-G.

OC (ppm)	$\rho_{r}$	$\sigma_{Peak}$ (MPa)	$\sigma_{Y}$ (MPa)	$\sigma_{Pl}\;$ (MPa)	E (MPa)	U (kJ/m^3^)	SEA (kJ/kg)
100	0.193	34.905	19.15	18.96	546.81	13,011.98	25.23
1000	0.191	36.705	15.475	19.03	587.67	13,144.81	25.78

**Table 5:** Mechanical properties of TPMS-D.

OC (ppm)	$\rho_{r}$	$\sigma_{Peak}$ (MPa)	$\sigma_{Y}$ (MPa)	$\sigma_{Pl}\;$ (MPa)	E (MPa)	U (kJ/m^3^)	SEA (kJ/kg)
100	0.33	33.23	29.35	27.344	654.105	14,081.45	15.95
1000	0.33	33.17	30	26.97	655.33	13,871.85	15.71

## Data Availability

The data that support the findings of this study are available from the corresponding author upon reasonable request.
